# Large tender abdominal aortic aneurysm presented with concomitant acute appendicitis: a case report

**DOI:** 10.1186/1757-1626-2-107

**Published:** 2009-01-30

**Authors:** Ahmad Al Samaraee, James McCaslin, Vish Bhattacharya

**Affiliations:** 1 Department of General Surgery, Queen Elizabeth Hospital, Queen Elizabeth Avenue, Sheriff Hill, Gateshead, Tyne & Wear, NE9 6SX, UK

## Abstract

**Introduction:**

The management of concurrently occurring abdominal aortic aneurysm and another intra-abdominal pathology is controversial and represents a difficult management problem for the surgeon. Most surgeons are reluctant to perform a second non vascular procedure at the time of the aneurysm repair because of the risk of graft infection. Some evidence suggests that the one-stage elective surgical treatment in selected patients with concomitant abdominal aortic aneurysm and other pathologies; especially Gastro-Intestinal malignancies, is safe with superior cost effectiveness. However, there is a major dilemma in the management patients with large aneurysm which require an urgent repair and presented with concomitant pathologies that carry a high risk of sepsis. In this case report, we describe an unusual presentation of a large aneurysm with concomitant Acute Appendicitis where both needed an urgent surgical intervention. To our best knowledge, there has been no similar case report published in literature.

**Case report:**

A 66 years old Caucasian male presented with a dual pathology of large abdominal aortic aneurysm and acute appendicitis. The diagnosis was confirmed by Computerized Tomography scan of his abdomen. He underwent a 2-stage operation; open Appendicectomy followed by open repair of his aneurysm to avoid the risk of graft infection. He had an uneventful recovery period with a full return to normal life.

**Conclusion:**

The incidence of patients with abdominal aortic aneurysm and coexistent intra-abdominal surgical pathology is increasing, and the surgical strategy for those patients remains controversial. There are not enough studies that looked directly into the management of large abdominal aortic aneurysm which require an urgent repair and presented with concomitant pathologies that carry a high risk of sepsis. In such situations, simultaneous operations should be avoided because of the risk of prosthetic graft infection and priority should be given to the symptomatic or most life threatening condition. The second pathology should be dealt with as soon as possible; preferably within the same admission. More studies are needed to look into this issue; however, this would be rather difficult because of the uncommon and complex nature of such presentations.

## Introduction

The management of concurrently occurring abdominal aortic aneurysm (AAA) and another intra-abdominal pathology is controversial. It is unclear whether to treat the aneurysm or the other pathology first, or both at the same time. Most clinicians agree that if one of the lesions is symptomatic it must be treated first [1]. Operating on the other pathology first might cause delay in operating on the AAA which could put the patient under an increasing risk of aneurysm rupture, particularly in large AAA. In addition, such operations might increase collagenase enzyme activity; hence in theory, might increase the possibility of AAA rupture [2]. While treatment of the AAA first exposes the patient to the risk of complications and progression of the other pathology, especially cancer. On the other hand, simultaneous surgery might lead to prosthetic graft infection. Various rates of grafts infection in synchronized surgery were reported in literature. In one of the largest series of combined open AAA and gastrointestinal surgery in 53 cases; 2 cases (i.e. 3.77%) were reported to have graft infection [1].

There are some reports of performing that one-stage elective surgical treatment in selected patients with concomitant AAA with gastric or colorectal cancer, with safety and superior cost effectiveness [3]. Moreover, there is some evidence showing that simultaneous elective Nephrectomy, Oophorectomy and Cholecystectomy can be performed during AAA repair without an apparent increased risk of graft infection [4]. In such cases, the key factor in the prevention graft infection is to operate on the AAA first with closure of the aneurysmal sac and posterior peritoneum securely over the aortic prosthesis [5]. Furthermore, endovascular aneurysm repair should always be considered, as this technique may have special advantages in patients with concomitant intra-abdominal disease [6]. This dilemma is even more evident in emergency situations where there is a potential for sepsis due to the nature of the simultaneous intra-abdominal pathology, combined with a high risk of AAA rupture due its size, where in both occasions an urgent operation is needed. In this case report, we describe the unusual presentation of a large AAA with concomitant Acute Appendicitis where both needed an urgent surgical intervention. To our best knowledge, there has been no similar case report published in literature.

## Case Report

A 66 years old Caucasian male presented with a 3 days history of nausea, vomiting, right lower abdominal pain and lower back pain. He had been complaining from upper abdominal discomfort for few months before his admission, this was treated as gastritis by his General Practitioner

The patient was known to have chronic obstructive air way disease (on inhalers). He was a smoker for years, who smoked about 25 cigarettes a day.

On clinical examination he was fully orientated, alert and not pale. His vital signs chart showed a temperature of 36.6°C, pulse of 85 and blood pressure of 153/61. Abdominal examination revealed a tender expansile mass in the upper abdomen with peritonism in the right iliac fossa.

**Figure 1:**
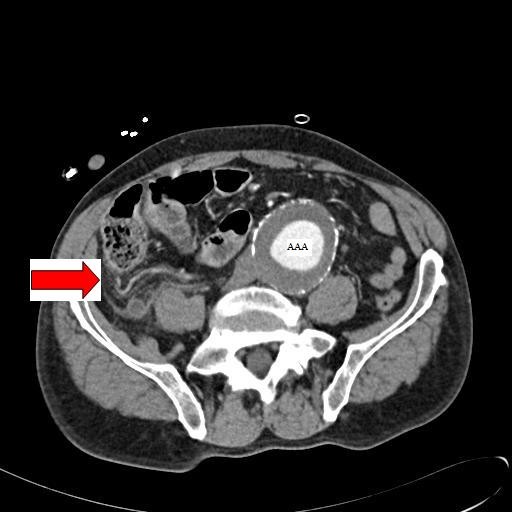
**Abdominal CT image with contrast taken on the day of admission prior to the open Appendicectomy; showing an inflamed appendix (*marked by the red arrow*) and the AAA**.

His blood tests showed elevated WCC of 13000 and CRP of 64 with normal Hb and other parameters. Due to his abdominal signs, an immediate abdominal Computerized Tomography (CT) scan was arranged. This showed a 6.3 cm infra-renal AAA which was not leaking. In addition, there were radiological signs of acute appendicitis (Fig. 1).

Due to the complex nature of the presentation and the risk of graft infection; a decision was made to perform a 2 stage-operation. Therefore, an open Appendicectomy was performed on the day of admission. This showed an inflamed appendix but no free fluid or pus. Appendicectomy was done then the abdominal cavity was washed out with1 Litre of warm normal saline. The patient had 3 doses in total of Intravenous (IV) antibiotics; Cefuroxime and Metronidazole (the first doses were given on induction). He had an uneventful post operative recovery; however, he was kept in the hospital for close observation and preparation of his AAA repair. The histopathology report showed features of acute appendicitis; with no other abnormality.

The possibility of endovascular repair of the AAA was excluded after discussion with an experienced endovascular radiologist; due to the very short neck of the AAA.

By the 10^th ^post-operative day the patient had appeared to have recovered completely from the appendicectomy, and his inflammatory markers were back to normal limits. So he underwent an open AAA repair with a 20 mm straight Dacron graft. He had one dose of IV Cefuroxime (1.5 g) on induction. There were no signs of peritoneal soiling or infection in the abdominal cavity. The aneurysmal sac was closed securely over the graft to decrease the risk of infection. He had an uneventful post-operative recovery period and he was discharged home 6 days after his AAA repair. He had been seen in outpatient clinic for routine follow up at 3 months, 6 months and 1 year and no problem was reported.

## Conclusion

The incidence of patients with AAA and coexistent intra-abdominal surgical pathology is increasing, and the surgical strategy for those patients remains controversial [7]. There is some evidence in favour of performing one-stage elective operations for intra-abdominal nonvascular surgical disorders, especially in properly selected patients with colorectal malignancies; nevertheless, such evidence remains spares as most of the published papers were case reports/series, or retrospective studies with no randomisation. The on hand literature suggests treating the most life threatening or symptomatic pathology first. In addition, endovascular grafting of the AAA can be a valuable tool in simplifying simultaneous treatment, or in staging the procedures with a very short delay [8].

However; and to our best knowledge, there are not enough studies that looked directly into the management of large AAA which require an urgent repair and presented with concomitant pathologies that carry a high risk of sepsis. In such scenarios, simultaneous operations should be avoided because of the risk of prosthetic graft infection. This case raises awareness of a rare combination of acute appendicitis and a large tender AAA.

## Abbreviations

AAA: Abdominal Aortic Aneurysm; CT: Computerized Tomography; IV: Intravenous; WCC: White Cell Count; CRP: C – Reactive Protein; Hb: Haemoglobin.

## Consent

Written informed consent was obtained from the patient for publication of this case report and any accompanying images. A copy of the written consent is available for review by the Editor-in-Chief of this journal.

## Competing interests

The authors declare that they have no competing interests.

## Authors' contributions

AALS was involved in the major parts of writing the paper and performing the literature search. He was also involved actively in the patients care in the pre and post operative period. JM contributed to the paper writing and to the literature search. He had also performed the open Appendicectomy and assisted in the AAA repair. VB is the responsible vascular surgeon who had set the management plan and the team leader. All authors have read the manuscript and agreed to its contents.
